# Caffeine Timing Improves Lower-Body Muscular Performance: A Randomized Trial

**DOI:** 10.3389/fnut.2020.585900

**Published:** 2020-11-23

**Authors:** Patrick S. Harty, Hannah A. Zabriskie, Richard A. Stecker, Brad S. Currier, Grant M. Tinsley, Kazimierz Surowiec, Andrew R. Jagim, Scott R. Richmond, Chad M. Kerksick

**Affiliations:** ^1^Exercise and Performance Nutrition Laboratory, School of Health Sciences, Lindenwood University, St. Charles, MO, United States; ^2^Energy Balance and Body Composition Laboratory, Department of Kinesiology & Sport Management, Texas Tech University, Lubbock, TX, United States; ^3^Mass Spectrometry Facility, Department of Chemistry & Biochemistry, Texas Tech University, Lubbock, TX, United States; ^4^Sports Medicine, Mayo Clinic Health System, Onalaska, WI, United States

**Keywords:** caffeine, performance, nutrient timing, strength, power

## Abstract

Little is known about the optimal time to consume caffeine prior to exercise to maximize the ergogenic benefits of the substance.

**Purpose:** To determine the optimal pre-exercise time interval to consume caffeine to improve lower-body muscular performance. A secondary aim was to identify the presence of any sex differences in responses to timed caffeine administration.

**Methods:** Healthy, resistance-trained males (*n* = 18; Mean±SD; Age: 25.1 ± 5.7 years; Height: 178.4 ± 7.1 cm; Body mass: 91.3 ± 13.5 kg; Percent body fat: 20.7 ± 5.2; Average caffeine consumption: 146.6 ± 100.3 mg/day) and females (*n* = 11; Mean ± SD; Age: 20.1 ± 1.6 years; Height: 165.0 ± 8.8 cm; Body mass: 65.8 ± 10.0 kg; Percent bodyfat: 25.8 ± 4.2; Average caffeine consumption: 111.8 ± 91.7 mg/day) participated in this investigation. In a randomized, double-blind, placebo-controlled, crossover fashion, participants consumed 6 mg·kg^−1^ caffeine or placebo solution at three time points: 2 h prior (2H), 1 h prior (1H), or 30 min prior (30M) to exercise testing. During three visits, caffeine was randomly administered at one time point, and placebo was administered at the other two time points. During one visit, placebo was administered at all three time points. Next, participants performed isometric mid-thigh pulls (IMTP), countermovement vertical jumps (CMVJ), and isometric/isokinetic knee extensor testing (ISO/ISOK).

**Results:** Caffeine administered at 1H significantly improved absolute CMVJ and ISO performance relative to placebo. Mean CMVJ jump height was significantly higher during 1H compared to 30M. However, only caffeine administered at 30M significantly improved absolute measures of isokinetic performance. Analysis of the pooled caffeine conditions revealed that muscular performance was more consistently augmented by caffeine in males compared to females.

**Conclusions:** Pre-exercise caffeine timing significantly modulated participant responses to the substance, with 1H exerting the most consistent ergogenic benefits relative to other time points, particularly compared to 2H. Male participants were found to respond more consistently to caffeine compared to female participants. These results suggest that active individuals can maximize the ergogenic effects of caffeine by consuming the substance ~1 h prior to the point when peak muscular performance is desired.

## Introduction

Caffeine (1,3,7-trimethylxanthine) is a commonly-consumed nutraceutical that has been shown to acutely improve the performance of endurance and resistance-based exercises (1–3). The ergogenic effects of caffeine have largely been attributed to its function as an adenosine receptor antagonist (4), as consumption has been shown to increase motor activity, neuromuscular coordination, and physiological arousal (5, 6). While much of the original research involving caffeine employed endurance models, a wide assortment of investigations have recently demonstrated that the consumption of caffeine in doses ranging from 3 to 9 mg·kg^−1^ body mass increases maximal isokinetic torque production (7, 8), isotonic force production (9, 10), muscular endurance (11–14), and power production (15–19) in active individuals. However, several investigations have also shown that acute caffeine consumption may exert little to no effect on the aforementioned parameters (11, 20–23), suggesting that substantial inter-individual variation exists in ergogenic responses to its ingestion. For example, several investigations failed to show significant mean differences in performance between caffeine and placebo, but reported the presence of a group of “responders” in the sample whose performance significantly benefited from ingestion of the substance (11, 15, 24, 25). Thus, it is possible that the inter-individual variation in responses to caffeine may result in non-significant differences in mean exercise performance when measured a limited number of times in a research setting, even though caffeine may be highly ergogenic for some participants (26). Indeed, a recent investigation (27) which compared the performance effects of caffeine relative to placebo on eight different occasions showed that even so-called ‘non-responders' experienced mild but detectable ergogenic effects following multiple bouts of performance testing. Caffeine habituation and the presence of certain genotype variations have been proposed as potential factors influencing the inter-individual variation in the response to caffeine and ergogenic outcomes (28, 29).

Another potential factor that may influence caffeine ergogenicity is the timing of caffeine administration prior to exercise testing, as previous investigators have suggested that the ergogenic effect of caffeine is maximized when peak plasma caffeine concentration coincides with the onset of physical activity (30) or when plasma caffeine concentration is still increasing during exercise (31, 32). Following ingestion, caffeine is completely absorbed in the stomach and small intestine, with plasma caffeine concentration increasing in a dose-dependent manner (33, 34). Though peak plasma caffeine concentration (C_max_) is typically reached within 30–60 min of oral ingestion of aqueous caffeine, C_max_ values have been observed 15–150 min post-consumption in subjects who consumed identical caffeine solutions (32, 33, 35). It is important to note that more rapid absorption merely results in a quicker onset of the effects of caffeine, but not necessarily their magnitude (36). In addition, variations in absorption kinetics have been attributed to differences in dosage (33), mode of caffeine administration (34, 36), dietary factors (37), and rate of gastric emptying (38). Regardless, because of the typical pharmacokinetic profile of caffeine, most researchers administer caffeine ~1 h prior to exercise testing (31, 39). However, such an approach may prevent research participants from undergoing exercise testing when plasma caffeine concentration is still increasing or near its peak. In addition, different forms of administration can greatly impact the time required to reach peak plasma caffeine concentrations, further complicating attempts to coincide C_max_ with the start of exercise testing (36).

To date, several investigations (30–32, 40) examining the impact of timed caffeine administration on endurance exercise performance have produced somewhat conflicting results. Bell and McLellan (30) first investigated the impact of caffeine timing in active, endurance-trained participants. The researchers administered either 5 mg·kg^−1^ body mass caffeine or placebo solution to the participants at 6, 3, and 1h prior to a cycle time-to-exhaustion (TTE) test at 80% maximal oxygen consumption (VO_2_max) and reported that TTE was significantly improved by all supplement conditions in caffeine non-users (defined as those who ingested <50 mg·day^−1^). However, regular caffeine users who typically ingested more than 300 mg caffeine only experienced appreciable performance benefits when caffeine was consumed either 1 or 3 h prior to exercise testing. A later study conducted by Ryan and colleagues (31) examined the impact of timed administration of caffeinated chewing gum on cycle time-trial performance in trained male cyclists who were moderate caffeine users (defined as ingesting <300 mg·day^−1^). In a crossover manner, the participants chewed caffeinated gum (containing 300 mg caffeine) at 120, 60, and 5-min time points prior to 15 min of cycling at 75% VO_2_max followed by a maximal 7 kJ·kg^−1^ cycle time-trial. The researchers reported that cycle time-trial performance was only enhanced when the caffeinated gum was administered 5 min before exercise. However, these results were in contrast to an earlier investigation by the same group (40), which found no effect of timed 200 mg caffeine gum administration (35 min before, 5 min before, or 15 min after exercise) on cycle TTE at 85% VO_2_max in physically-active males who were moderate caffeine users. Finally, Skinner et al. (32) found that well-trained subjects (VO_2_max > 60 mL·kg^−1^·min^−1^) who consumed 6 mg·kg^−1^ body mass caffeine 1 h prior to a 40 km cycle time-trial completed the time-trial significantly faster relative to placebo. However, when an identical pre-exercise dose of caffeine was consumed at an individualized, pre-exercise time point which would evoke peak plasma caffeine concentration at the start of exercise, significant performance improvements were not identified.

More information is needed regarding the optimal time to administer caffeine prior to exercise, particularly as no studies have been conducted to examine the effect of timed caffeine administration on maximal force production, power production, and muscular endurance. The identification of an optimal time interval to consume caffeine prior to resistance exercise has significant implications for active individuals, practitioners, and the dietary supplement industry, as a wide array of products and drinks containing caffeine are marketed to be consumed before exercise (41, 42). In addition, limited information is available regarding the effects of caffeine supplementation in female participants, though preliminary information suggests that males and females experience similar effects following acute consumption (43). Thus, the purpose of this investigation was to examine the effects of three pre-exercise caffeine timing strategies on force production, power production, and muscular endurance in a cohort of resistance-trained males and females. A secondary aim of this study was to identify the presence of any sex differences in responses to timed caffeine administration. It was hypothesized that all caffeine conditions would improve performance, but that caffeine administered 1 h prior to exercise would result in the greatest performance benefit compared to caffeine administered at other time points. It was also hypothesized that performance outcomes would be similar between men and women.

## Materials and Methods

### Experimental Overview

**Figure 1:**
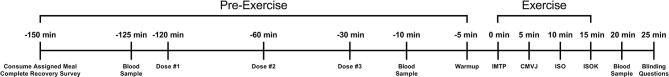
Experimental design. CMVJ, Counter-movement vertical jump; IMTP, Isometric mid-thigh pull; ISO, Isometric knee extensor assessment; ISOK, Isokinetic knee extensor assessment; Min, minutes.

This randomized, double-blind, placebo-controlled, crossover study consisted of five visits to the laboratory. Following completion of an Institutional Review Board-approved (Lindenwood University IRB-19-L0029, Approval date: 9/17/2018), written informed consent document, eligible participants completed an initial familiarization session consisting of a body composition assessment via dual-energy x-ray absorptiometry (DXA) and practice trials of all performance tests, which included isometric mid-thigh pulls, countermovement vertical jumps, and knee extensor testing using an isokinetic dynamometer. Because participant expectations of the effects of caffeine have been shown to influence responses to the substance (44), a verbal explanation of the effects of caffeine on muscular performance was provided to participants at the end of the familiarization session. Following the familiarization session, all participants completed four identical testing sessions separated by at least 48 h (Figure 1). Participants abstained from exercise, caffeine, and alcohol for 24 h and fasted from all calorie-containing foods and beverages for at least 8 h prior to all five visits to the laboratory. In addition, participants recorded their dietary intake for 48 h prior to their second visit and were instructed to replicate their recorded intake during the 2 days before all remaining study visits. Upon arrival to the laboratory for each testing session, participants completed a brief customized sleep status questionnaire, consumed a standardized meal, and donated a venous blood sample to allow for baseline assessment of plasma caffeine content. Following the baseline blood sample and at three designated time points, participants consumed solutions containing either caffeine (6 mg·kg^−1^ of body mass) or placebo (maltodextrin) at 120, 60, and 30 min prior to exercise testing, in randomized order. During the three experimental conditions, participants consumed caffeine at a single pre-determined time point and placebo at all other time points. During the placebo condition, a placebo was consumed at all three ingestion points. Between the consumption of each supplement solution, the participants remained seated and were allowed to read, talk, or use technology. Following supplement consumption and 10 min prior to performance testing, a second blood sample was collected to assess pre-exercise plasma caffeine concentration. Immediately after their second blood collection, a 5-min standardized warmup was completed and participants then completed an isometric mid-thigh pull test, countermovement vertical jumps, an isometric knee extension test using an isokinetic dynamometer, and an isokinetic knee extensor fatigue protocol using an isokinetic dynamometer. Within 5 min of completing exercise testing, a third venous blood sample was collected to determine post-exercise plasma caffeine concentration. Total testing time was ~20 min.

### Subjects

Healthy, resistance-trained males [*n* = 18; Mean ± SD (Range); Age: 25.1 ± 5.7 years; Height: 178.4 ± 7.1 cm; Body mass: 91.3 ± 13.5 kg; Percent body fat: 20.7 ± 5.2% fat; Average absolute caffeine consumption: 146.6 ± 100.3 mg·day^−1^ (0–282 mg·day^−1^); Average relative caffeine consumption: 1.6 ± 1.1 mg·kg body mass^−1^·day^−1^ (0–3.7 mg·kg body mass^−1^·day^−1^)] and females [*n* = 11; Mean ± SD (Range); Age: 20.1 ± 1.6 years; Height: 165.0 ± 8.8 cm; Body mass: 65.8 ± 10.0 kg; Percent bodyfat: 25.8 ± 4.2 % fat; Average absolute caffeine consumption: 111.8 ± 91.7 mg·day^−1^ (0–300 mg·day^−1^); Average relative caffeine consumption: 1.8 ± 1.5 mg·kg body mass^−1^·day^−1^ (0–4.7 mg·kg body mass^−1^·day^−1^)] were recruited to participate in this study. All participants reported at least 6 months of structured resistance-training experience and had engaged in resistance training at least twice per week for at least 45 min per session at the time of enrollment in the study. Participants were excluded if they consumed more than 300 mg caffeine per day, were not between 18 and 45 years of age, or were currently being treated for any metabolic, cardiac, musculoskeletal, pulmonary, renal, immunological, psychological, or metabolic disease or taking any medications (prescription or over-the-counter) that might influence the physiological responses to caffeine or resistance exercise at the time of enrollment. In addition, current tobacco users and those who could not fit on testing equipment were excluded from participation. All participants reported habitual caffeine consumption <300 mg·day^−1^ via completion of a brief, customized caffeine habits questionnaire and were instructed to maintain their supplementation, dietary, and training regimens throughout the study protocol. Finally, all female participants were required to confirm that they were not pregnant or trying to become pregnant via written questionnaire. All testing sessions for male participants were separated by at least 48 h. Eumenorrheic and oligomenorrheic female participants underwent their first testing session as close as possible to 14 days after the start of their most recent menstrual cycle, and follow-up testing sessions were scheduled in 1-week intervals following their initial visit. Amenorrheic female participants were scheduled in 1-week intervals with no respect to menstrual phase. Female participants who reported using oral contraceptives or intrauterine devices were not excluded from participation. In addition, it is important to note that caffeine appears to have relatively similar ergogenic effects across different phases of the menstrual cycle, as shown by Romero-Moraleda et al. (45).

### Procedures

#### Anthropometric and Body Composition Assessments

All body composition and anthropometric measurements were obtained during the initial familiarization session. Body mass was determined using a digital balance (BWB-627A Class III, Tanita Corp, Inc., Tokyo, Japan) and height was assessed using a stadiometer (HR-200, Tanita Corp, Inc., Tokyo, Japan). Body composition was determined via dual-energy x-ray absorptiometry (DXA) (Hologic Discovery A, Hologic Inc., Bedford, MA). Participants were positioned on the scan table according to manufacturer instructions. All scans were analyzed by the same trained researcher using the manufacturer-provided software package (Hologic APEX Version 4.5.3) and the NHANES correction factor. The test-retest reliability of these DXA procedures in our laboratory has been previously shown to be excellent (ICC ≥ 0.996) using the same device and a similar population.

#### Sleep Status and Blinding Questionnaires

During all testing sessions, participants completed a brief sleep status questionnaire immediately upon arrival to the laboratory. Participants reported how many hours of sleep they had the night prior to the testing visit and rated their perceived sleep quality on a scale from 0 to 10. In addition, the average amount of sleep per night during the entire week prior to the testing visit was reported. Following completion of each testing visit, participants reported which supplement condition they thought was administered to them during the visit and answered a single question assessing the presence of acute caffeine withdrawal.

#### Supplementation Protocol

Over the course of this investigation, participants completed all four supplemental conditions in a randomized, double-blind, placebo-controlled, crossover manner. The order of the supplemental conditions was determined using a computerized random number generator (46). During each data collection session, a solution of 175 mL water containing either caffeine or placebo and flavored with a non-caloric sweetener (Crystal Light, Kraft Foods, Inc., Chicago, IL) was provided to participants at three time points: 120 min prior to exercise testing (2H), 60 min prior to testing (1H), and 30 min prior to testing (30M). During three of the sessions, a dose of anhydrous caffeine equivalent to 6 mg·kg^−1^ body mass was administered at one time point and placebo was administered at the other two time points. For the placebo condition, placebo was administered at all three time points. The caffeine was chosen to be administered in liquid form due to the wide array of caffeinated drinks (coffee, tea, soda, energy drinks) and powdered supplements designed to be mixed with water (multi-ingredient pre-workout supplements) that are commonly consumed by active individuals to augment exercise performance (41, 47). To aid in blinding, the pre-prepared caffeine conditions consisted of individual body mass-specific doses of powdered anhydrous caffeine mixed with ~3 g of pure maltodextrin powder (Carbo Gain, Now Foods, Bloomingdale, IL) and were stored in sachets. Similarly, the placebo sachets contained ~4 g of maltodextrin powder, making them visually identical to the caffeine sachets. Prior to each study visit, the powdered supplemental conditions were mixed with 175 mL of water and ~2.5 g of non-caloric sweetener until all powder was completely dissolved. To prevent participants from detecting differences in flavor between the placebo and caffeine conditions, three different flavors of the non-caloric sweetener were used during each visit, and one flavor was added to each of the three drinks in a randomized fashion. Participants were required to completely drink the supplement solution within 1 min of the specified time point. In addition, participants rinsed the container the beverage was stored in and consumed the remaining liquid to ensure that no residue was left behind.

#### Dietary Intake Standardization

Participants recorded all calorie-containing foods and fluids consumed during the 48 h prior to arriving for the first data collection session. Each food log packet contained images and instructions detailing how to accurately assess portion sizes of foods and beverages. Following the initial testing session, participants were provided a copy of the food log and instructed to replicate their dietary intake during the 2 days preceding each subsequent study visit. However, compliance with these instructions was not tracked, which is a potential limitation of our approach. Upon arrival to the laboratory for each testing session, participants were provided with a small, standardized, carbohydrate-rich meal (170 kcal, 1.5 g fat, 40 g carbohydrate, 1 g protein) which was consumed 150 min prior to exercise testing. This meal was provided to reduce gastrointestinal distress resulting from consumption of caffeine following an overnight fast. However, it is important to note that this meal may have influenced participants' rates of gastric emptying, resulting in greater heterogeneity during the resultant performance tests.

#### Performance Testing

##### Isometric Mid-thigh Pull (IMTP) testing

Following a 5-min standardized warmup consisting of dynamic bodyweight movements such as walking lunges, squats, and leg swings, participants performed four exercise tests to assess lower-body force production, power production, muscular endurance, and fatigue resistance. Immediately after the warmup, participants performed an isometric mid-thigh pull testing protocol consisting of a single 5-s trial repetition followed by three 5-s maximal pulls, with 30 s of rest between repetitions. An adjustable-height horizontal handle was attached via chain to a computerized load cell sampling at 150 Hz (iLoad Pro Digital USB, Loadstar Sensors, Fremont, CA) to measure the peak tensile force generated during each mid-thigh pull repetition. The knurled handle was positioned approximately halfway between the subjects' patella and inguinal fold so that the body position of the participants when gripping the handle at this height closely resembled the start of the second pull phase of a barbell clean (48). The height of the handle was recorded during the familiarization session for each participant and replicated during all testing sessions. Participants were verbally encouraged to pull upward as hard and fast as possible, as this cue has been demonstrated to result in optimal force production during mid-thigh pull testing (49). A double-overhand grip was used during all repetitions. Peak force production and average force production of the three trials were recorded. Isometric mid-thigh pull assessments have been shown to be strongly related to athletic performance outcomes such as strength (48, 50), sprint performance (50, 51), and agility performance (50). Importantly, this type of test does not require the same level of technical skill and has reduced risk of injury compared to dynamic tests of maximal strength such as one-repetition maximum tests (52).

##### Countermovement Vertical Jump (CMVJ) testing

Following 2 min of rest after the mid-thigh pull assessment, a countermovement vertical jump testing protocol was completed. Participants stood in a neutral athletic stance with each foot positioned at shoulder width on a separate force platform sampling at 1,000 Hz (PS-2141, Pasco, Roseville, CA). Following a single trial repetition, each participant performed three maximal-effort countermovement vertical jumps with 10 s of rest in between each attempt. Participants bent their knees to a partially-flexed position, and without pausing, rapidly jumped upward as high as possible before landing on the force plates in an athletic position. Vertical jump height was calculated using the time-in-air method, according to the following equation. In this equation, *g* is equal to 9.81 m·s^−2^, *t* is equal to the time that the participant was in the air while jumping from the force plates, and jump height is expressed in meters.

$$\begin{array}{r} \text{Jump Height }\left(\text{m}\right)=1/2\text{g}{\left(\frac{\text{t}}{2}\right)}^{2} \end{array}$$

Participants completed all jumps with their hands on their hips, as alterations in the center of gravity resulting from arm swinging have been shown to dramatically increase vertical jump performance (53). Peak jump height and average jump height of the three trials were recorded.

##### Isometric and isokinetic knee extensor testing

Following 5 min of rest, knee extensor testing was completed using an isokinetic dynamometer (Biodex System 3, Biodex Medical Systems, Shirley, NY). Participants were positioned on the dynamometer in accordance with lower-body testing procedures provided by the manufacturer. The axis of rotation of the knee joint of the participants' self-reported dominant leg was aligned with the axis of the dynamometer unit and the participant was secured using seat and leg restraints during all testing procedures. Participant positioning was recorded during the familiarization session and replicated during all study visits. Following positioning and calibration of the dynamometer, participants performed a maximal knee extension trial repetition lasting 5 s. Next, three maximal voluntary contractions of 5 s duration were performed by participants, with 30 s of rest between each repetition. All isometric knee extension repetitions were completed at an angle of 60° flexion below horizontal. Peak isometric torque production and the average peak torque production across the three repetitions were recorded.

After 5 min of rest, isokinetic knee extensor testing of the dominant leg was completed using identical positioning procedures according to the methods of Thorstensson and Karlsson (54). Following the completion of five maximal trial repetitions, participants performed 50 maximal knee extension repetitions at 180°/s, extending from a flexed position (90°) to a fully extended knee angle (0°). During the flexion phase of each repetition, participants lowered their legs passively. This testing protocol was completed in ~50 s. Peak torque, average peak torque, total work completed during the test, work completed during the first third of the test, work completed during the last third of the test, and work fatigue percentage were recorded. Immediately following completion of the knee extensor testing protocol, rating of perceived exertion (on a scale from 0 to 10) and perceived muscular pain (on a scale from 0 to 10) were collected by investigators.

#### Venipuncture and Plasma Caffeine Analysis

Venous blood samples were obtained from a forearm vein located in the antecubital region using standard phlebotomy procedures at three time points: 125 min prior to exercise testing, 10 min before exercise, and immediately following exercise. All venous blood samples were collected using heparinized vacutainer tubes and centrifuged at 20°C for 10 min at 3,500 RPM. Plasma samples were transferred into labeled microcentrifuge tubes and frozen at −80 °C for analysis of plasma caffeine concentrations. Plasma caffeine levels were quantified via high-performance liquid chromatography using the methods described by Alvi and colleagues (55). Thermo Scientific Ultimate 3000 HPLC system equipped with the Diode Array Detector and thermostated column oven was used. Data acquisition was collected and processed using Chromeleon software. Thermo Scientific column Acclaim 120, C18 3 um 120 A 3 × 150 m mm at a flow rate of 0.4 mL/min.

#### Statistical Analyses

Descriptive statistics were calculated using standard statistical methods. Shapiro–Wilk tests were employed to assess the normality of all outcome measures. When data was identified as skewed or non-normal, log_10_ transformations were completed. When log_10_ transformations did not improve model assumptions for non-normal data, non-transformed data was reported. The presence of outliers in the raw data was examined using box plots. An additional analysis was completed with all outliers removed and no material changes in our reported outcomes were identified. As a result, all outliers were retained. To determine the combined effect of all caffeine conditions on performance, caffeine conditions for the total sample and for each sex were pooled and compared against the placebo condition using paired samples *t*-tests. To determine the presence of any main and interaction effects for sleep status as well as all absolute performance outcomes, multiple 4 × 2 (supplement condition by sex) ANOVAs were performed with condition as a within-subjects factor and sex as a between-subjects factor. ANOVAs with condition as a within-subjects factor were employed to determine main effects and between-condition differences in performance outcomes within each sex. A 4 × 3 (supplement condition by time) repeated measures ANOVA was employed to analyze plasma caffeine levels at each collected time point. When the sphericity assumption was violated, Greenhouse-Geisser corrections were applied. Partial eta squared ($\eta_{\text{P}}^{2}$) was calculated as SS_Effect_/(SS_Effect_ + SS_Error_). These values can be cautiously interpreted to denote small, medium, or large effects when exceeding benchmarks of 0.0099, 0.0588, and 0.1379, respectively (56). Differences in absolute ordinal data for each sex and for the entire sample (Pain, Perceived sleep quality, and RPE) were determined via Friedman tests, and *post-hoc* comparisons of ordinal data were completed using Wilcoxon signed rank tests. The Bonferroni correction was applied to all pairwise comparisons to control the familywise error rate. Total and sex-specific ordinal data from the three caffeine conditions were pooled and compared against the placebo condition using Wilcoxon Signed Rank tests. Effect sizes (Cohen's *d*) were calculated for all statistically-significant (*p* < 0.05) differences between conditions and were interpreted as trivial (0–0.19), small (0.20–0.49), medium (0.50–0.79) or large (0.80 or greater) (57). For all statistical tests, a significance threshold of *p* < 0.05 was used. All analyses were completed using Microsoft Excel 2016 (Microsoft Corp., Redmond, WA) and IBM SPSS Statistics (Version 25.0, IBM Corp., Armonk, NY). Figures were generated via Prism (Version 8.1.0, GraphPad Corp., San Diego, CA) and R (R Core Team, Vienna, Austria).

## Results

### Sleep Status

A repeated measures ANOVA revealed no significant interaction effect (*p* = 0.649) or significant main effects (*p* < 0.806) for sleep duration during the night prior to testing. Similarly, the interaction effect (*p* = 0.661) and main effects (*p* < 0.560) for average sleep during the week prior to testing were not found to be significant. However, a significant (*p* = 0.012) between-condition difference in perceived sleep quality was detected using a Friedman test. *Post-hoc* analysis was completed via multiple Wilcoxon signed-rank tests, and a Bonferroni correction was applied to control the familywise error rate. It was found that perceived sleep quality during the 2H condition was significantly lower than during 1H (Z = −2.655, *p* = 0.008).

### Performance Outcomes

#### Isometric Mid-thigh Pull Force Production (IMTP)

**Figure 2:**
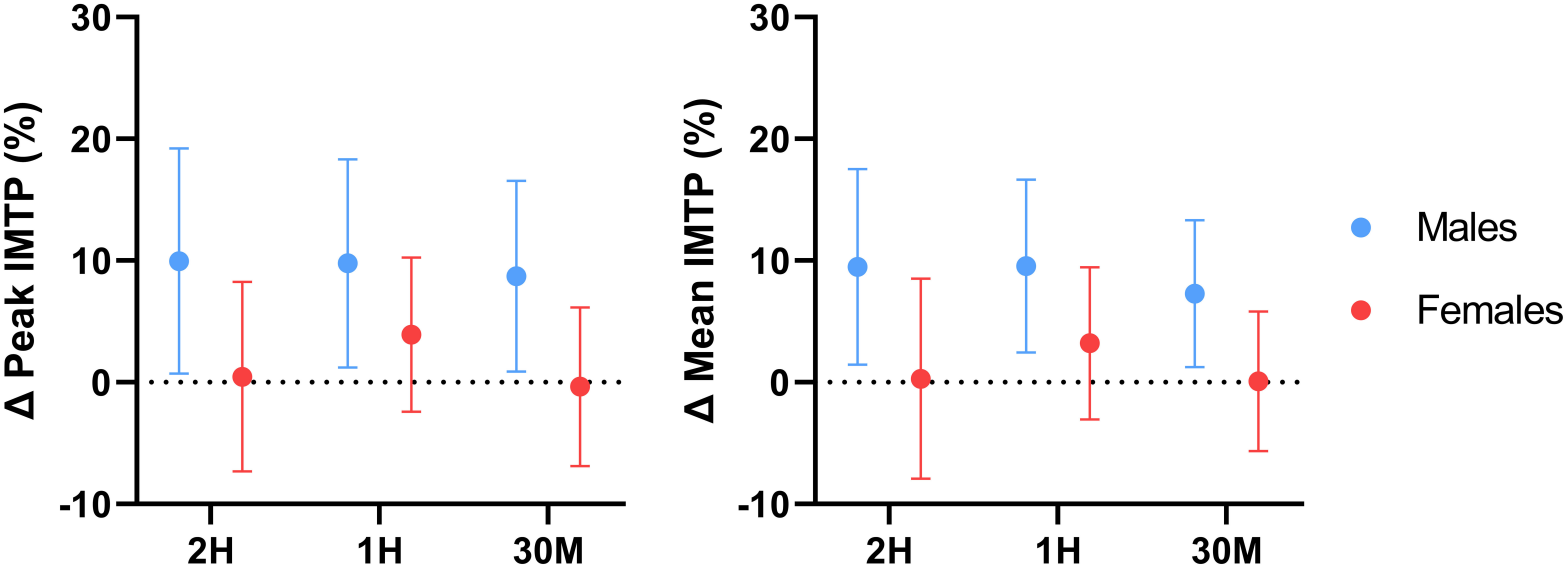
Effect of caffeine timing on isometric mid-thigh pull performance. 2H, 2Hours; 1H, 1Hour; 30M, 30 Minutes; IMTP, Isometric mid-thigh pull; %, Percentage; Δ, Delta. Line plots represent sex-specific group means and 95% confidence intervals for all delta values relative to the placebo value.

Repeated measures ANOVA revealed no differences in absolute mean and peak IMTP performance for the total sample and within both sexes (Table 1). Sex-specific response figures comparing the percent change in IMTP performance from baseline for each condition are depicted in Figure 2. Paired-samples *t*-tests revealed that peak (*p* = 0.019; *d* = 0.21) and mean (*p* = 0.015; *d* = 0.21) IMTP performance were significantly greater than placebo in the pooled caffeine conditions for the total sample. Peak (*p* = 0.011; *d* = 0.39) and mean (*p* = 0.008; *d* = 0.38) IMTP performance in the pooled caffeine conditions were significantly greater than placebo in males. No differences between the pooled caffeine conditions and placebo were identified in females (*p* < 0.963).

**Table 1:** Influence of caffeine timing on performance outcomes.

**Variable**	**Group**	**2 H (*d*)**	**1 H (*d*)**	**30 Min (*d*)**	**Placebo**	***p*****-value (**$\eta_{\text{P}}^{2}$**)**		
						**Condition**	**Sex**	***I***
Peak IMTP (N)	Male	1,770.5 ± 411.4	1,772.5 ± 416.8	1,766.3 ± 448.5	1,621.9 ± 337.7	0.011 (0.195)	<0.001 (0.390)	0.096 (0.075)
	Female	1,177.0 ± 349.9	1,198.8 ± 242.6	1,151.4 ± 242.3	1,177.2 ± 337.9	0.707 (0.045)		
	Total	1,545.4 ± 482.0	1,554.9 ± 454.7	1,533.1 ± 485.1	1,453.2 ± 397.7	0.097 (0.075)		
Mean IMTP (N)	Male	1,669.7 ± 379.7	1,678.9 ± 395.8	1,652.1 ± 411.7	1,534.3 ± 313.8	0.010 (0.198)	<0.001 (0.407)	0.130 (0.067)
	Female	1,098.7 ± 307.7	1,119.5 ± 238.4	1,085.3 ± 225.1	1,098.7 ± 290.4	0.830 (0.029)		
	Total	1,453.1 ± 448.2	1,466.7 ± 437.8	1,437.1 ± 446.5	1,369.1 ± 369.1	0.077 (0.080)		
Peak CMVJ Jump height (m)	Male	0.39 ± 0.09^*^(0.34)	0.39 ± 0.08^*^^†^ (0.30)	0.37 ± 0.08	0.36 ± 0.08	0.001 (0.348)	0.012 (0.211)	0.071 (0.091)
	Female	0.30 ± 0.06^*^ (0.32)	0.31 ± 0.06^*^ (0.46)	0.31 ± 0.07	0.28 ± 0.06	0.033 (0.334)		
	Total	0.36 ± 0.09^*^ (0.29)	0.360 ± 0.08^*^ (0.31)	0.35 ± 0.08^*^ (0.21)	0.33 ± 0.08	<0.001 (0.279)		
Mean CMVJ jump height (m)	Male	0.37 ± 0.08^*^ (0.31)	0.38 ± 0.08^*^^†^ (0.34)	0.36 ± 0.08	0.35 ± 0.09	<0.001 (0.425)	0.011 (0.215)	0.543 (0.024)
	Female	0.29 ± 0.06^*^ (0.38)	0.30 ± 0.06^*^ (0.50)	0.29 ± 0.06^*^ (0.35)	0.27 ± 0.06	<0.001 (0.547)		
	Total	0.34 ± 0.08^*^ (0.29)	0.35 ± 0.08^*^^†^ (0.35)	0.34 ± 0.08^*^ (0.20)	0.32 ± 0.08	<0.001 (0.436)		
Isometric peak torque (N·m)	Male	269.5 ± 78.6	270.3 ± 59.8^*^ (0.37)	270.4 ± 65.2^*^ (0.36)	247.6 ± 62.4	0.011 (0.195)	<0.001 (0.403)	0.485 (0.030)
	Female	169.5 ± 49.6	182.2 ± 42.8	173.9 ± 42.9	163.1 ± 37.9	0.040 (0.239)		
	Total	231.5 ± 84.0	236.9 ± 68.7^*^ (0.31)	233.8 ± 74.2^*^ (0.26)	215.5 ± 68.0	0.002 (0.162)		
Mean isometric peak torque (N·m)	Male	256.5 ± 77.3	257.3 ± 58.3^*^ (0.43)	257.8 ± 63.9^*^ (0.41)	232.7 ± 55.7	0.001 (0.291)	<0.001 (0.381)	0.249 (0.049)
	Female	161.2 ± 49.2	175.4 ± 42.0	167.8 ± 41.0^*^ (0.30)	155.8 ± 39.4	0.029 (0.257)		
	Total	220.3 ± 81.9	226.2 ± 65.8^*^ (0.35)	223.6 ± 71.1^*^ (0.30)	203.5 ± 62.3	<0.001 (0.225)		
Isokinetic peak torque (N·m)	Male	191.8 ± 38.0	193.9 ± 39.3	193.0 ± 37.3	184.5 ± 36.3	0.029 (0.161)	<0.001 (0.526)	0.422 (0.034)
	Female	116.7 ± 32.8	123.5 ± 26.0	124.3 ± 25.8	117.4 ± 27.3	0.048 (0.229)		
	Total	163.3 ± 51.3	167.2 ± 48.9	167.0 ± 47.3^*^ (0.17)	159.0 ± 46.5	0.006 (0.143)		
Mean isokinetic peak torque (N·m)	Male	120.8 ± 25.0	121.9 ± 25.6	124.7 ± 26.5^*^ (0.23)	118.7 ± 25.5	0.006 (0.214)	<0.001 (0.506)	0.366 (0.038)
	Female	74.7 ± 17.5	78.8 ± 14.4	78.9 ± 14.7	76.6 ± 14.8	0.193 (0.144)		
	Total	103.3 ± 31.74	105.5 ±30.4	107.3 ± 31.9^*^ (0.14)	102.7 ± 30.1	0.006 (0.143)		
Total isokinetic work (J)	Male	7,061.9 ± 1097.4	7,044.5 ± 1141.6	7,171.1 ± 1052.1	6,841.7 ± 984.3	0.07 (0.128)	<0.001 (0.605)	0.821 (0.011)
	Female	4,615.2 ± 914.3	4,763.3 ± 832.7	4,777.4 ± 891.0	4,548.8 ± 951.8	0.455 (0.082)		
	Total	6,133.8 ± 1,577.8	6,179.2 ± 1,519.2	6,263.1 ± 1,533.8	5,972.0 ± 1,481.1	0.051 (0.091)		
Isokinetic work first third (J)	Male	3,341.3 ± 666.4	3,341.7 ± 626.5	3,398.5 ± 596.4^*^ (0.51)	3,095.4 ± 587.4	0.004 (0.231)	<0.001 (0.522)	0.420 (0.034)
	Female	2,141.9 ± 531.9	2,221.5 ± 426.0	2,224.7 ± 476.1	2,089.3 ± 535.1	0.262 (0.126)		
	Total	2,886.3 ± 849.4	2,916.8 ± 780.5^*^ (0.27)	2,953.3 ± 795.5^*^ (0.31)	2,713.8 ± 747.4	0.004 (0.154)		
Isokinetic work last third (J)	Male	1,425.1 ± 278.7	1,426.7 ± 234.0	1,444.9 ± 221.4	1,500.7 ± 234.7	0.213 (0.084)	<0.001 (0.547)	0.640 (0.020)
	Female	995.1 ± 165.6	1,023.0 ± 197.3	1,011.8 ± 182.1	1,018.6 ± 188.5	0.921 (0.016)		
	Total	1,262.0 ± 319.5	1,273.6 ± 294.8	1,280.6 ± 295.5	1,317.8 ± 320.6	0.437 (0.033)		
Isokinetic work fatigue (%)	Male	56.1 ± 10.8^*^ (0.53)	56.6 ± 6.9^*^ (0.67)	56.8 ± 6.8	50.0 ± 12.0	0.002 (0.244)	0.377 (0.029)	0.615 (0.022)
	Female	51.7 ± 10.5	53.3 ± 8.2	53.8 ± 6.6	49.4 ± 12.1	0.093 (0.190)		
	Total	54.4 ± 10.7^*^ (0.41)	55.3 ± 7.5^*^ (0.56)	55.7 ± 6.8^*^ (0.61)	49.8 ± 11.8	0.002 (0.189)		

All data are presented as Mean ± Standard Deviation. P-values and measures of effect size (partial eta squared) are reported for all main effects (condition, sex) and interaction effects (condition x sex) associated with each outcome. Effect sizes (Cohen's d) are provided for all statistically-significant (p < 0.05) differences between conditions, as determined by Bonferroni post-hoc analysis. All effect sizes are presented relative to the within-group (Male, Female, or Total) placebo trial. CMVJ, Countermovement vertical jump; I, Interaction Effect; IMTP, Isometric mid-thigh pull; J, Joules; RPE, Rating of perceived exertion; m, meters; N, Newtons; N-m, Newton-meters; W, Watts; %, Percentage; $\eta_{\text{P}}^{2}$, Partial eta squared.

* Denotes significant within-group difference (p < 0.05) from placebo.

† *Denotes significant within-group difference (p < 0.05) from 30 Min*.

#### Countermovement Vertical Jump Height (CMVJ)

**Figure 3:**
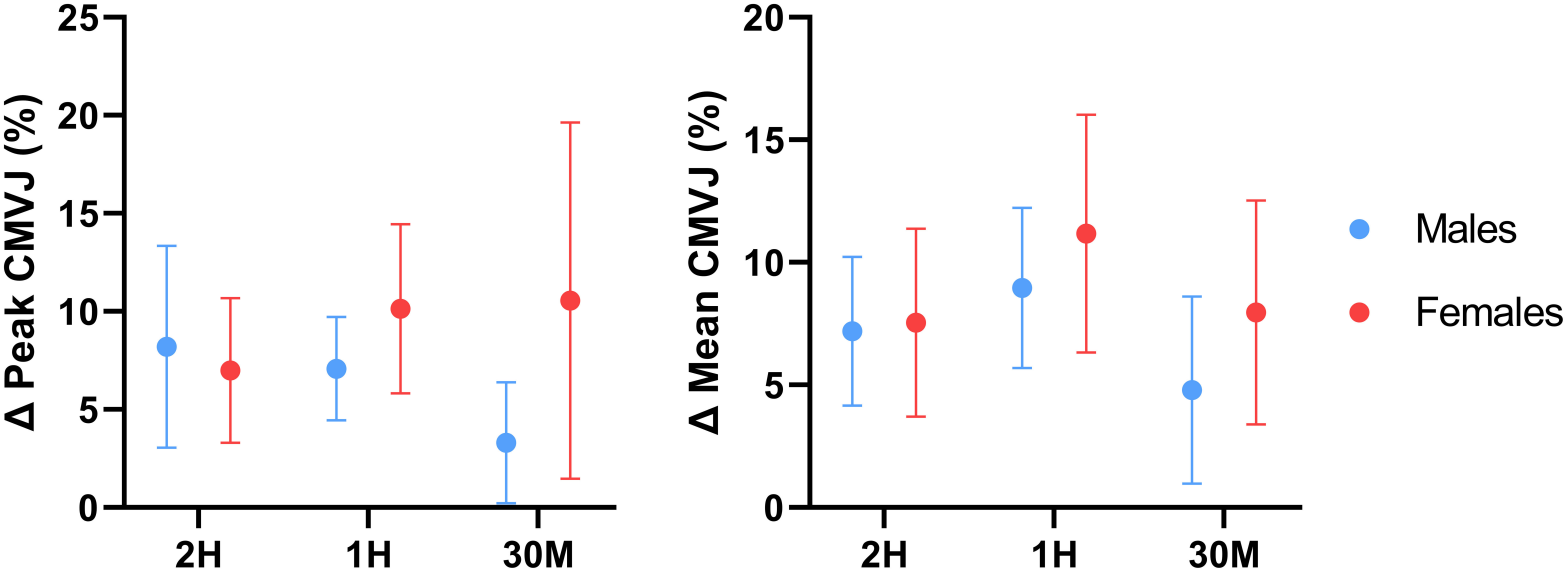
Effect of caffeine timing on counter-movement vertical jump performance. 2H, 2 Hours; 1H, 1 Hour; 30M, 30 Minutes; CMVJ, Counter-movement vertical jump; %, Percentage; Δ, Delta. Line plots represent sex-specific group means and 95% confidence intervals for all delta values relative to the placebo value.

Repeated measures ANOVA of the absolute performance results of the total sample revealed that peak and mean CMVJ performance were significantly improved relative to placebo by all caffeine conditions, with 1H producing the greatest magnitude of ergogenic effect for both outcomes, followed by 2H and 30M (Table 1). In addition, mean jump height during 1H was found to be significantly higher than in the 30M condition for the total sample. Repeated measures ANOVA of sex-specific results revealed that peak jump height was significantly greater than placebo in the 2 and 1H conditions for both males and females, though a significant difference between 1H and 30M was only detected in males. Similar results were observed for mean jump height; caffeine administered at 2 and 1H improved performance relative to placebo in both sexes. However, mean CMVJ performance during 1H was significantly >30M in males only, and performance during the 30M condition was significantly greater than placebo in female participants only. Sex-specific response figures comparing the percent change in CMVJ performance from baseline for each condition are depicted in Figure 3. Paired samples *t*-tests revealed that peak (*p* < 0.001; *d* = 0.28) and mean (*p* = 0.001; *d* = 0.28) CMVJ jump height were significantly greater than placebo in the pooled caffeine conditions for the total sample. Peak (*p* = 0.001; *d* = 0.26) and mean (*p* < 0.001; *d* = 0.28) jump height in the pooled caffeine conditions were significantly greater than placebo in males. This pattern was mirrored in females; peak (*p* < 0.001; *d* = 0.42) and mean (*p* < 0.001; *d* = 0.41) CMVJ performance were significantly augmented relative to placebo.

#### Isometric Knee Extensor Torque Production (ISO)

**Figure 4:**
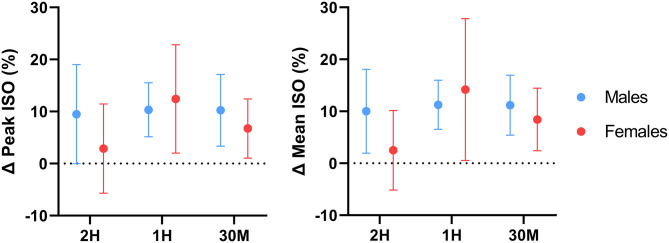
Effect of caffeine timing on isometric knee extensor performance. 2H, 2 Hours; 1H, 1 Hour; 30M, 30 Minutes; ISO, Isometric knee extensor performance; %, Percentage; Δ, Delta. Line plots represent sex-specific group means and 95% confidence intervals for all delta values relative to the placebo value.

Analysis of absolute performance data for the total sample revealed that peak and average peak torque production were significantly augmented relative to placebo during the 1H and 30M conditions, with the greatest magnitude of difference observed during the 1H condition (Table 1). Sex differences in absolute peak and absolute mean peak torque production were also detected; caffeine administered at 1H and 30M significantly improved peak and mean peak isometric knee extensor torque in males. However, only mean peak torque production was greater than placebo in females during the 30M condition. Sex-specific response figures comparing the percent change in ISO performance from baseline for each condition are depicted in Figure 4. Peak (*p* < 0.001; *d* = 0.26) and mean peak (*p* < 0.001; *d* = 0.30) isometric torque production for the total sample were significantly greater than placebo in the pooled caffeine conditions. Peak (*p* < 0.001; *d* = 0.35) and mean peak (*p* = 0.001; *d* = 0.40) ISO performance in the pooled caffeine conditions were significantly greater than placebo in males. Peak (*p* = 0.016; *d* = 0.30) and mean peak (*p* = 0.019; *d* = 0.30) ISO torque production in the pooled caffeine condition were also significantly greater than placebo in females.

#### Isokinetic Knee Extensor Torque Production (ISOK)

**Figure 5:**
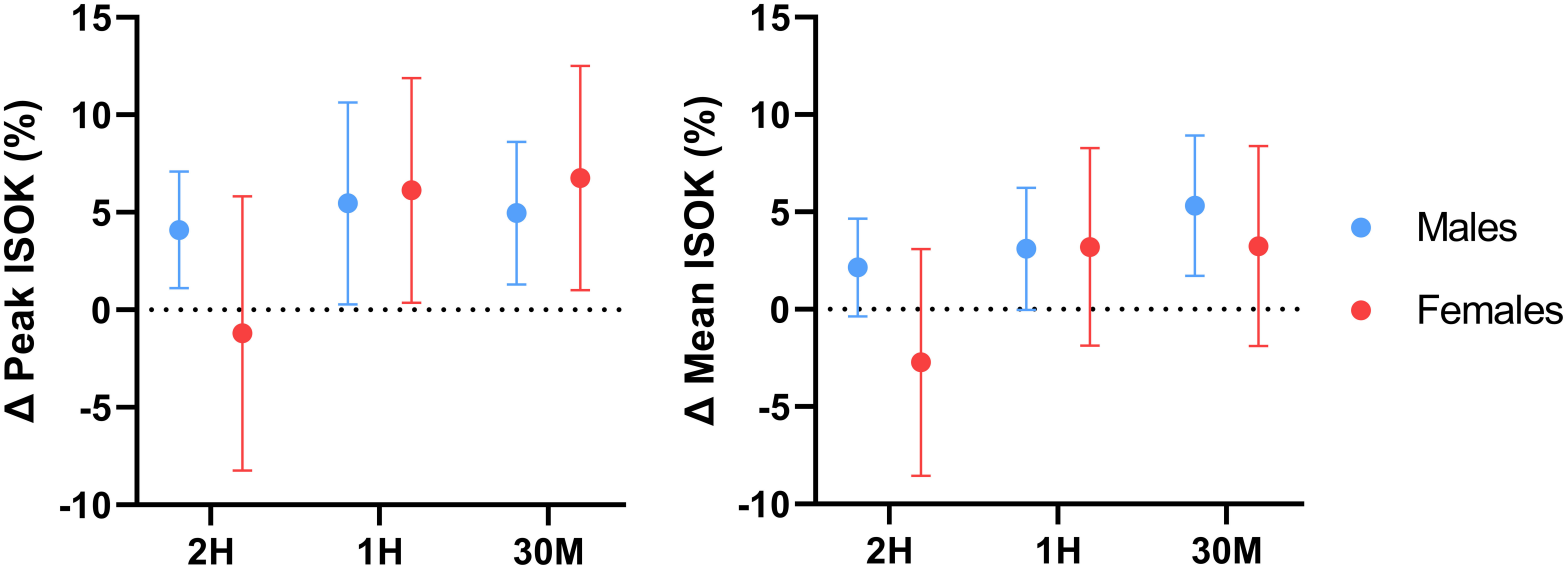
Effect of caffeine timing on isokinetic knee extensor performance. 2H, 2 Hours; 1H, 1 Hour; 30M, 30 Minutes; ISOK, Isokinetic knee extensor performance; %, Percentage; Δ, Delta. Line plots represent sex-specific group means and 95% confidence intervals for all delta values relative to the placebo value.

Peak (*p* = 0.048; *d* = 0.15) but not mean peak (*p* = 0.455) isokinetic torque was significantly augmented by the pooled caffeine conditions in females. Absolute mean and mean peak isokinetic performance for the total sample were only increased relative to placebo in the 30M condition (Table 1). Analysis of the absolute performance data for each sex revealed that only mean isokinetic peak torque was significantly improved by the 30M condition in males only. Sex-specific response figures comparing the percent change in ISOK performance from baseline for each condition are depicted in Figure 5. Peak (*p* = 0.001; *d* = 0.14) and mean peak (*p* = 0.008; *d* = 0.09) isokinetic torque production were significantly greater than placebo in the pooled caffeine conditions for the total sample. Peak (*p* = 0.008; *d* = 0.23) and mean peak (*p* = 0.01; *d* = 0.15) ISOK performance in the pooled caffeine condition were significantly greater than placebo in males.

No between-condition differences in total isokinetic work were found for the total sample or for each sex. However, isokinetic work completed during the first third of the test was significantly augmented during the 1H and 30M conditions in the total sample and in males only. No differences in any work variable were identified in the female participants. Work fatigue percentage for the total sample was significantly higher than placebo during all three caffeine conditions. Work fatigue percentage was significantly greater than placebo in both the 2H and 1H conditions in males, while no between-condition differences were detected for females.

### Plasma Caffeine Concentration

**Figure 6:**
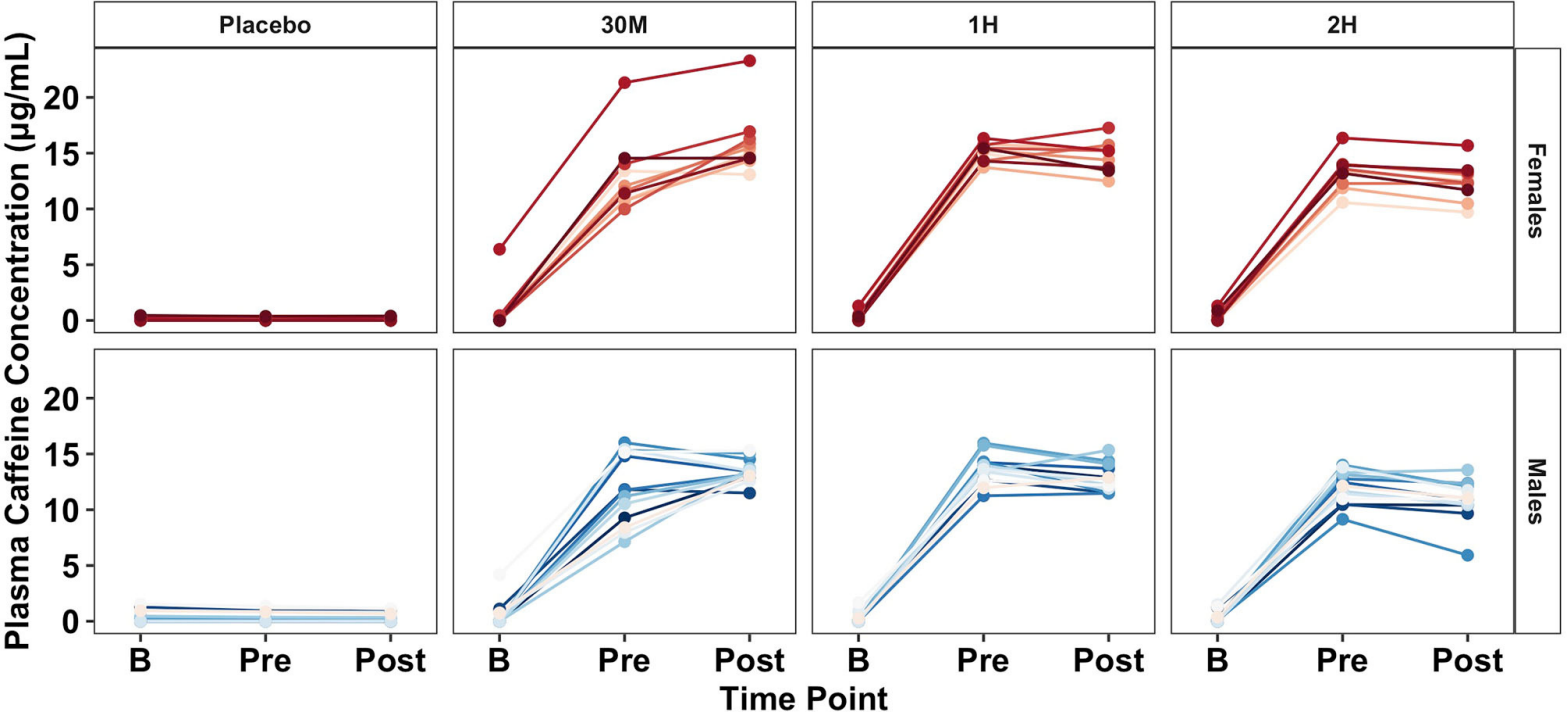
Effect of caffeine timing on plasma caffeine concentration. 2H, 2 Hours; 1H, 1 Hour; 30M, 30 Minutes; B, Baseline Time Point; Pre, Pre-Exercise Time Point; Post: Post-Exercise Time Point; P, Placebo; μg/mL, micrograms per milliliter. Center bars represent sex-specific group means.

Complete plasma caffeine concentration data was available for 13 male and 10 female participants (Mean ± SD; Age: 21.9 ± 2.9 years; Height: 173.1 ± 10.8 cm; Body mass: 81.6 ± 18.0 kg; Percent body fat: 23.4 ± 6.2%; Average caffeine consumption: 124.0 ± 97.3 mg/day). Significant main (*p* < 0.001) and interaction (*p* < 0.001) effects were identified. No significant between-condition differences in plasma caffeine concentration were found at baseline (Figure 6). Pre-exercise caffeine concentration was significantly higher in the 1H condition (14.28 ± 1.36 μg·mL^−1^) relative to all other conditions (*p* < 0.031). Post-exercise caffeine concentration was highest in the 30M condition (14.54 ± 2.31 μg·mL^−1^), which was significantly higher (*p* < 0.001) than the 2H condition (11.58 ± 1.88 μg·mL^−1^) and placebo (0.23 ± 0.34 μg·mL^−1^).

### Subjective Responses

Wilcoxon signed rank tests revealed that subjective ratings of RPE (Z = −2.221, *p* = 0.026) but not Pain (Z = −0.474, *p* = 0.636) were significantly decreased relative to placebo in the pooled caffeine condition for the total sample. RPE (Z = −0.569, *p* = 0.569) and Pain (Z = −0.979, *p* = 0.328) were not different from placebo in males. However, ratings of RPE (Z = −2.288, *p* = 0.022) but not Pain (Z = −0.052, *p* = 0.959) during the pooled caffeine conditions were significantly lower than placebo in females. Friedman tests detected no significant between-condition differences for absolute or delta RPE and Pain ratings for the total sample, as well as within each sex.

Participants were able to correctly identify the supplement condition they received during 64.7% of all testing visits. The effectiveness of the blinding is in line with the results of a previous investigation (60% correct identification) which employed an identical dose of encapsulated caffeine (20). Six out of 29 participants reported the presence of perceived ergolytic caffeine withdrawal symptoms during at least one testing visit. These symptoms were reported during 6.9% of all visits, with 50% occurring during the placebo condition.

## Discussion

The purpose of this investigation was to determine the optimal pre-exercise time to consume caffeine to maximize muscular performance and to identify any sex differences in caffeine ergogenicity and responses to caffeine timing. The results of this study indicate that, in the context of a laboratory setting, acute caffeine supplementation improved lower-body force production, power production, isometric torque production, and isokinetic torque production in resistance-trained men and women. Furthermore, the timing of caffeine administration was found to significantly influence the ergogenic effects of the substance, as caffeine consumed 1 h prior to exercise testing produced the most consistent ergogenic effect across all performance outcomes. Finally, divergent performance results were identified between sexes, suggesting that resistance-trained males and females may respond in different ways to acute caffeine supplementation.

Analysis of the absolute performance data collected by this investigation revealed that the timing of caffeine administration significantly modulated the ergogenic effects of the substance. Caffeine consumed 1 h prior to exercise improved absolute mean and peak CMVJ and ISO performance to a greater degree than the other two supplement conditions, as indicated by effect size analysis (Table 1). In addition, absolute mean CMVJ jump height was significantly greater during the 1H condition compared to 30M, further supporting the efficacy of caffeine consumed 1 h prior to exercise testing compared to other time points. Similarly, analysis of the subgroup of subjects with complete plasma caffeine concentration data suggests that peak pre-exercise plasma caffeine concentration was significantly modulated by the timing protocols employed in the present investigation. Based on these results, it appears that pre-exercise plasma caffeine concentration is maximized by consuming caffeine administered in liquid form 1 h prior to exercise. However, post-exercise caffeine concentration was largest in the 30M condition, particularly compared to 2H and P. This general pattern is mirrored by the performance data, suggesting that caffeine timing strategies to improve performance should center on maximizing plasma caffeine concentrations at either the start of exercise or when peak performance is needed.

Though direct comparisons between the present investigation and other caffeine timing studies are difficult due to differences in exercise modality and mode of caffeine administration, the identification of timing-related performance benefits in the present sample mirrors the findings of several earlier investigations, albeit with a different mode of exercise (30, 32). For example, Skinner et al. (32) noted that 6 mg·kg^−1^ caffeine in capsule form significantly improved 40 km cycle time trial performance when administered 1 h prior to exercise but had no ergogenic effect when administered at 120 or 150 min before testing. Similarly, Bell and McLellan (30) reported that caffeine administered at 1 or 3H but not 6H prior to exercise significantly improved performance during cycle time-to-exhaustion testing in caffeine users. It is important to note that only the 30M condition in the present investigation resulted in significant improvements in absolute isokinetic torque production. However, because the entire muscular performance testing battery took ~20 min to complete, the onset of isokinetic testing occurred ~45-50 min after ingestion of the 30M condition. Thus, it is possible that the results of isokinetic testing more closely reflect the ergogenic effects of caffeine administered 1 h prior to testing had the isokinetic test been performed first in the testing protocol. These results suggest that a more nuanced approach to pre-exercise caffeine timing may allow for targeted improvement of exercise performance during an extended training session or athletic competition. In short, consuming caffeine ~1 h before the exact time that peak performance is desired may be a superior timing strategy compared to consuming caffeine 1 h prior to the start of exercise or competition.

Comparisons between the pooled caffeine condition and placebo indicate that acute pre-exercise caffeine consumption significantly improved lower-body force production, power production, and muscular endurance in the resistance-trained individuals recruited by this investigation. These findings support the ergogenic utility of acute caffeine supplementation and align with several earlier investigations which reported significant increases in knee extensor isometric force production (58, 59), isokinetic torque production (7, 8, 60–62), and total isokinetic work completed (63) following the ingestion of moderate doses of caffeine (5–7 mg·kg^−1^). In addition, the effect sizes of the improvement in isometric (*d* = 0.24–0.30, *p* < 0.001) and isokinetic (*d* = 0.09–0.14, *p* < 0.008) knee extensor strength found in the pooled caffeine condition are similar to the overall standardized mean difference (SMD) for the caffeine-mediated improvement in knee extensor MVC strength (SMD = 0.37, *p* = 0.0002) found by Warren et al. (64) in a 2010 meta-analysis as well as to the SMD (0.19, *p* = 0.004) for isokinetic knee extensor strength reported by Grgic and Pickering (65) in a 2019 meta-analysis. Similarly, the ergogenic benefit of acute caffeine consumption on lower-body power production demonstrated by this investigation mirror the results of Bloms et al. (19), who reported a significant effect of acute caffeine consumption (5 mg·kg^−1^) on countermovement jump height in a mixed sex cohort of resistance-trained collegiate athletes. The lack of any demonstrable effect of caffeine supplementation on perceived pain during repeated isokinetic contractions also reflects the results of several investigations (62, 66) which found no effect of caffeine on perceived pain during isokinetic knee extensor contractions at 180°·s^−1^. However, acute caffeine supplementation significantly lowered participants' subjective rating of perceived exertion during isokinetic exercise, in accordance with previous investigations (67). In addition, it is important to note that a degree of inter-individual variability in the responses to caffeine was observed in the present investigation, which is particularly evident when examining the confidence intervals of the figures displaying percent change from placebo (Figures 2–5). These responses were retained in the dataset because they represented true participant performance responses. This determination was made after removing all of them from the original analysis (with them included), which resulted in the same statistical outcomes being identified as when the outliers were included. The changes in these outcomes could largely be explained by comparatively poor performance in the placebo condition relative to the three experimental conditions, or particularly excellent peak performance in one or more conditions. These differences could potentially be caused by differences in motivation, warm-up efficacy, motivation, self-effort, and exercise execution between the four conditions. In addition, other variables such as menstrual phase could have impacted our results (68). However, all of these variables were controlled, as stated in the methods section. It should be emphasized that these figures showing percent change from placebo are entirely intended for descriptive purposes, as all analyses performed in the present investigation used absolute performance data. Finally, to determine if potential outliers resulting from variable performance data had any impact on the conclusions of this investigation, a sensitivity analysis was performed in which all outliers (measurements from 1 to 2 individual participants per outcome variable) were removed and the data were re-analyzed. Following re-analysis with outliers removed for each outcome variable, all main results were unchanged, further supporting the conclusions made using the original analytical approach.

The results of this investigation suggest the presence of sex-specific differences in responses to caffeine, as muscular performance was augmented more consistently after ingestion of the substance in males compared to females (Table 1). In addition, paired-samples *t*-tests showed that peak and mean IMTP force production were significantly enhanced relative to placebo in the pooled caffeine conditions for males, while no differences relative to placebo were detected in females. Finally, non-parametric analysis of the pooled caffeine conditions revealed that RPE during isokinetic exercise was significantly decreased by caffeine consumption in female participants only. These results are particularly meaningful, as a limited number of caffeine investigations have recruited female participants and even fewer have examined sex-specific responses to caffeine in a mixed-sex cohort (43). One possible explanation for the sex-specific responses observed in this investigation may be due to the influence of sex hormones on gastric emptying, as several investigations have shown that elevated levels of progesterone result in slower rates of gastric emptying (69, 70). The presence of sex-specific responses to caffeine aligns with the results observed by Sabblah et al. (71), who administered caffeine (5 mg·kg^−1^) to resistance-trained males and females 1 h prior to bench press one-repetition maximum (1RM) testing and bench press repetitions to fatigue at 40% 1RM. Though only a trend (*p* = 0.059) was detected for total weight lifted (TWL) during the muscular endurance test, males increased TWL by ~24% while the TWL by females did not appreciably change (<1% change). The authors concluded that the female participants' response to caffeine was of lesser magnitude than male participants, which aligns with the results of the present investigation. However, it is important to note that Sabblah et al. (71) employed a single-blind study design and did not attempt to control for menstrual phase, thus limiting the generalizability of their results.

In conclusion, the present investigation revealed for the first time that pre-exercise caffeine timing significantly modulated the ergogenic impact of the substance on lower-body muscular performance, identified significant differences in responses to acute caffeine supplementation between sexes, and further supported the utility of acute caffeine consumption to improve lower-body muscular performance. Based on the results of this study, it appears that caffeine consumed 1 h prior to exercise exerts the most consistent ergogenic benefits relative to other time points. However, those seeking to improve lower-body muscular performance during extended training sessions or competitions should consume caffeine ~1 h prior to the time when peak performance is most needed, rather than 1 h prior to the start of exercise/competition. These results apply to a wide variety of active individuals who consume caffeine prior to resistance exercise or prior to activities which require lower-body force and power production. Additional research is needed to determine the effect of caffeine timing on upper-body muscular performance as well as sport-specific performance in other populations. Future research should also investigate the impact of caffeine timing using lower doses of caffeine, multiple doses for longer-duration bouts of exercise, other forms of caffeine administration, as well as potential genetic influences on optimal caffeine timing.

## Data Availability Statement

The raw data supporting the conclusions of this article will be made available by the authors, without undue reservation.

## Ethics Statement

The studies involving human participants were reviewed and approved by Lindenwood University Institutional Review Board. The patients/participants provided their written informed consent to participate in this study.

## Author Contributions

CK and PH designed the study. PH, HZ, RS, and BC assisted in data collection under the direction of CK. PH, GT, and CK contributed to statistical analysis. KS analyzed all biological samples. PH, AJ, SR, and CK contributed to the initial manuscript draft. All authors reviewed and approved the final version of the manuscript.

## Conflict of Interest

The authors declare that the research was conducted in the absence of any commercial or financial relationships that could be construed as a potential conflict of interest.
